# Prognostic and Predictive Value of Three DNA Methylation Signatures in Lung Adenocarcinoma

**DOI:** 10.3389/fgene.2019.00349

**Published:** 2019-04-24

**Authors:** Yanfang Wang, Haowen Deng, Shan Xin, Kai Zhang, Run Shi, Xuanwen Bao

**Affiliations:** ^1^Ludwig-Maximilians-Universität München, Munich, Germany; ^2^Chair for Computer Aided Medical Procedures and Augmented Reality, Technical University Munich, Munich, Germany; ^3^Institute of Molecular Toxicology and Pharmacology, Helmholtz Center Munich, German Research Center for Environmental Health, Neuherberg, Germany; ^4^Department of Cardiology, Sir Run Run Shaw Hospital, Zhejiang University School of Medicine, Hangzhou, China; ^5^Institute of Radiation Biology, Helmholtz Center Munich, German Research Center for Environmental Health, Neuherberg, Germany; ^6^Technical University Munich (TUM), Munich, Germany

**Keywords:** LUAD, DNA methylation, regularized logistic regression, recursive feature elimination, LASSO Cox regression, metastasis

## Abstract

**Background:** Lung adenocarcinoma (LUAD) is the leading cause of cancer-related mortality worldwide. Molecular characterization-based methods hold great promise for improving the diagnostic accuracy and for predicting treatment response. The DNA methylation patterns of LUAD display a great potential as a specific biomarker that will complement invasive biopsy, thus improving early detection.

**Method:** In this study, based on the whole-genome methylation datasets from The Cancer Genome Atlas (TCGA) and several machine learning methods, we evaluated the possibility of DNA methylation signatures for identifying lymph node metastasis of LUAD, differentiating between tumor tissue and normal tissue, and predicting the overall survival (OS) of LUAD patients. Using the regularized logistic regression, we built a classifier based on the 3616 CpG sites to identify the lymph node metastasis of LUAD. Furthermore, a classifier based on 14 CpG sites was established to differentiate between tumor and normal tissues. Using the Least Absolute Shrinkage and Selection Operator (LASSO) Cox regression, we built a 16-CpG-based model to predict the OS of LUAD patients.

**Results:** With the aid of 3616-CpG-based classifier, we were able to identify the lymph node metastatic status of patients directly by the methylation signature from the primary tumor tissues. The 14-CpG-based classifier could differentiate between tumor and normal tissues. The area under the receiver operating characteristic (ROC) curve (AUC) for both classifiers achieved values close to 1, demonstrating the robust classifier effect. The 16-CpG-based model showed independent prognostic value in LUAD patients.

**Interpretation:** These findings will not only facilitate future treatment decisions based on the DNA methylation signatures but also enable additional investigations into the utilization of LUAD DNA methylation pattern by different machine learning methods.

## Introduction

Lung cancer is the leading cause of cancer-related mortality globally, causing over a million deaths a year (Genome Atlas Research Network., 2014; Jemal et al., 2018). There are two clinical types, one is the aggressive subtype small cell lung cancer and the other is non-small cell lung cancer (Hankey et al., 1999). Non-small cell lung cancer is histologically classified into four major subtypes by pathological and molecular characteristics: adenocarcinoma, large cell lung cancer, squamous cell lung cancer, and other types (Ettinger et al., 2010). Adenocarcinoma is the most common histological subtype of non-small cell lung cancer. Tobacco smoking is the major cause of lung adenocarcinoma (Toh et al., 2006). However, with the decrease in the number of smokers in many countries, the occurrence of LUAD in non-smokers has increased (Genome Atlas Research Network., 2014).

An accurate diagnosis of LUAD is one precondition to achieve a better treatment effect. Although the Mayo Clinic stage, size, grade, and necrosis (SSIGN) score, as well as the University of California Integrated Staging System can help improve the accuracy of the prognosis (Travis et al., 2011), the outcomes of patients with similar clinical characteristics or integrated systems scores still differ. Molecular characteristics may provide an indication for predicting the LUAD prognosis and response to therapy, thus offering great potential for improving individual treatment. Moreover, molecular characterization-based methods do not generally require bulk tissue samples, which can improve the patients' tolerance and reduce unnecessary operation steps. Among all the molecular characteristics, DNA methylation of CpG sites plays a crucial role in epigenetic regulation by reducing the activity of a DNA segment and repressing gene transcription (Jones, 2012; Du et al., 2015; Schübeler, 2015). DNA methylation is associated with carcinogenesis by repressing the expression of the tumor suppressor gene and promoting the expression of oncogenes (Herman et al., 1995; Schübeler, 2015; Vizoso et al., 2015; Klutstein et al., 2016). Hence, the cancer tissues have a distinct DNA methylation pattern compared to normal tissues. More importantly, unlike somatic genetic mutations in tumor tissues, DNA methylation patterns are inherently reversible changes and can therefore be promising targets for drug treatments (Ramchandani et al., 1999). Using DNA methylation signatures can help us make a better prognosis and predict the treatment response, thus prolonging the patients' survival.

Machine learning is a novel method to learn concept from data, which will help researchers discover the hidden insights. Based on DNA methylation patterns, machine learning techniques are developed and used to design models for precise classification and accurate prediction in medicine. In this study, we evaluated the possibility of DNA methylation signatures in identifying LUAD lymph node metastasis, differentiating between tumor tissue and normal tissue and predicting the OS of LUAD patients by applying TCGA whole-genome methylation datasets to several machine learning methods. Our results showed robust classifier effects with the AUC of both classifiers achieving values close to one for identifying lymph node metastasis and differentiating between tumor tissue and normal tissue. Cross-validation was applied to prevent overfitting. The LASSO Cox regression model was used to evaluate the patients' OS. Risk scores from the LASSO Cox model were combined with other clinicopathological risk factors to generate a nomogram to predict the prognosis and help the doctors to manage LUAD patients.

## Methods

### Data Source

The DNA methylation files and patients' information were obtained from Xena (https://xenabrowser.net/). Complete clinical, molecular, and histopathological data-sets are available at the TCGA website (https://portal.gdc.cancer.gov/).

### Feature Selection for DNA CpG Sites

We formulate critical methylation identification as a feature selection problem. Each CpG site is treated as a feature here and our goal is to find out which features are important for different tasks.

### Variance Based Filtering

Variance is the squared deviation of the data from its mean, showing the spread of numbers. It is an important characteristic that reflects the distribution and discriminability of a feature. The variance σ of an observed sample sequence of a given feature {*x*_1_, *x*_2_, …, *x*_*i*_, …, *x*_*N*_} is computed by averaging across the squared difference of each value to the mean μ.

$$\begin{array}{l} \sigma^{2}=\frac{\sum {\left(x_{i}-\mu \right)}^{2}}{N}\\ \mu =\frac{\sum x_{i}}{N} \end{array}$$

In general, a larger variance σ means a wider distributed and more separable feature space, which facilitates training a classifier to find class boundaries. On the other hand, variance σ is positively correlated with information entropy *E*, meaning that more information could be obtained with a larger variance σ. When σ is small, all the data is compressed and provides insufficient information for a classifier, so that we would avoid features with a small variance by setting a minimum threshold to filter out the indiscriminate features.

$$\begin{array}{l} E=-\int P\left(x\right)\log P\left(x\right)dx \end{array}$$

### Regularized Logistic Regression Model

Logistic regression is a widely applied and useful statistical, non-linear model for predicting a binarized outcome based on a sequence of independent features. Assuming we have a general linear regression model *y*, which satisfies

$$\begin{array}{l} y=\overset{N}{\underset{i=0}{\sum }}\beta_{i}x_{i} \end{array}$$

where *x*_*i*_ stands for the *i*-th feature and β_*i*_ is the correspondent coefficient. Since there is no constraint on the range of β_*i*_ and *x*_*i*_, there is no maximum or minimum limit for *y*, i.e., *y* ∈ [−∞, ∞]. Consider a standard logistic function

$$\begin{array}{l} f\left(x\right)=\frac{1}{1+e^{-x}} \end{array}$$

This could map an input space from an infinite [−∞, ∞] to a finite [0, 1]. By combining the linear regression model with the logistic function, we obtain the logistic regression model

$$\begin{array}{l} y=\frac{1}{1+e^{-\sum_{i=1}^{N}\beta_{i}x_{i}}} \end{array}$$

By thresholding *y* with threshold *t*, we obtain the binarized output.

$$\begin{array}{l} o=\{\begin{array}{c} 1,y\geq t\\ 0,y<t \end{array} \end{array}$$

Note that, regularization term can be compounded with a logistic regression model, to force the learned coefficients to be sparser and more resistant to overfitting, which is highly beneficial for feature selection as well. We term the logistic regression model with regularization term as “Regularized logistic regression model.”

### Recursive Feature Elimination

Recursive feature elimination (RFE) adopts a brute-force and recursive way of undermining important features. Given a pre-defined model, which weighs all the features internally, RFE recursively uses the set of features to train the model and discard features that are the least important for the model (e.g., small weights) and repeats the training with the remaining features. This operation keeps recycling until certain expectations are reached, such as the maximum number of expected features *N*_exp_. The process is described in Algorithm 1.

**Algorithm 1:** Recursive Feature Elimination.

**INPUT**: a set of features *S* = {*f*_1_, *f*_2_, …, *f*_*i*_, …, *f*_*n*_}, expected feature number *N*_exp_
**OUTPUT**: a set of kept features
*S*_*best*_ = {*f*_*s*_1__, *f*_*s*_2__, …, *f*_*s*_*i*__, …, *f*_*s*_*n*__}
**WHILE** size (*S*) > *N*_exp_ **DO**
Train a model with the set of features in *S* Get the coefficients for the features learned from the classification model Prune features with small coefficients *S*_*non* − *important*_ Update feature set *S* = *S* − *S*_*non*−*important*_
**END WHILE**
Keep the final set of features as the set of most important features *S*_*best*_ = *S*__

### Cox Regression

Cox regression, also called Proportional Hazards Regression, is a survival analysis model. It can be used to analyze relationships between different features and the survival time. The Cox model is based on the proportional hazards condition, which assumes that features have a proportional relationship to the exponential change of hazard. Thus, the model is formulated as a multiplication of a baseline hazard function with a sole time variable *t*, and an exponential function of the linear combination of all of the features as an input. Given a set of *n* samples {(***X***_*i*_, *Y*_*i*_, *s*_*i*_) | 0 ≤ *i* ≤ *n, i* ∈ ***R***}, where *X*_*i*_ = (*x*_*i*0_, *x*_*i*1_, …, *x*_*ik*_) and stands for the *i*-th sample of all the *k* features, *Y*_*i*_ is the observation time and *s*_*i*_ is the survival status, the hazard function is

$$\begin{array}{l} H_{i}\left(t\right)=H_{0}\left(t\right)e^{X_{i}^{T}\beta } \end{array}$$

***β*** = (β_**0**_,β_1_, …, β_*k*_) is the coefficient vector weighing the contribution of the features. The partial likelihood of all the samples is

$$\begin{array}{l} L\left(\beta \right)=\overset{n}{\underset{i=1}{\prod }}L_{i}\left(\beta \right)\\ =\overset{n}{\underset{i=1}{\prod }}\frac{H_{i}\left(Y_{i}\;|\;X_{i}\right)}{\sum_{j:Y_{j}\geq Y_{i}}H_{i}\left(Y_{i}|\;X_{j}\right)}\\ =\overset{n}{\underset{i=1}{\prod }}\frac{e^{X_{i}^{T}\beta }}{\sum_{j:Y_{j}\geq Y_{i}}e^{X_{j}^{T}\beta }} \end{array}$$

By penalizing -log (*L*(**β**)), the optimal **β** could be uncovered.

### LASSO Regularization

LASSO (Least Absolute Shrinkage and Selection Operator) is an important regularization in many regression analysis methods. The concept behind LASSO is that an L1-norm is used to penalize the weight of the model parameters. Assuming a model has a set of parameters {*w*_0_, *w*_1_, …, *w*_*n*_}, the LASSO regularization can be written as

$$\begin{array}{l} \lambda \cdot \overset{n}{\underset{i=0}{\sum }}\|w_{i}\|{\;}_{1} \end{array}$$

It can be also expressed as a constraint to the targeted objective function

$$\begin{array}{l} \sum \|Y-Y^{*}\|{\;}_{2}\;,\;s.t.\;\|w_{i}\|{\;}_{1}<\;t \end{array}$$

An important property of the LASSO regularization term is that it can force the parameter values to be 0, thus generating a sparse parameter space, which is a desirable character for feature selection.

### Workflow of the Coding Process

When it came to selecting the methylation features for the metastasis and tumor identification problems, we first used variance-based filtering to eliminate some of the least important CpG sites, and to decrease the computation for the following Regularized Logistic Regression Model and RFE. To avoid model overfitting and bias in the feature selection, cross validation was used in the following stages. The dataset was evenly divided into 5-folds, and further feature selections were conducted by applying Logistic Regression Model and RFE, following the standard pipeline of cross validation.

When predicting the OS of LUAD patients, we built the Cox proportional hazard regression model with LASSO regularization. 5-fold cross-validation was applied to avoid the overfitting. We plotted the plots in R software (R Foundation for Statistical Computing, Vienna, Austria. Version 3.4.3) and Python (Python Software Foundation. Python Language Reference, version 3.7).

## Results

### Preparation of LUAD DNA Methylation Datasets

LUAD DNA methylation data and corresponding clinical data were downloaded from Xena (https://xenabrowser.net/) (Cline et al., 2013). After removing samples without a survival status and normalization, a total of 478 samples were analyzed in the present study (Supplementary File 1). The datasets included 409 samples for the recognition of metastasis, 428 samples for the recognition of tumor from normal tissue, and 446 samples for the prediction of OS [(Supplementary Files 2–(4].

### Identification of 3616-CpG-Based Signature for the Recognition of Metastasis

**Figure 1:**
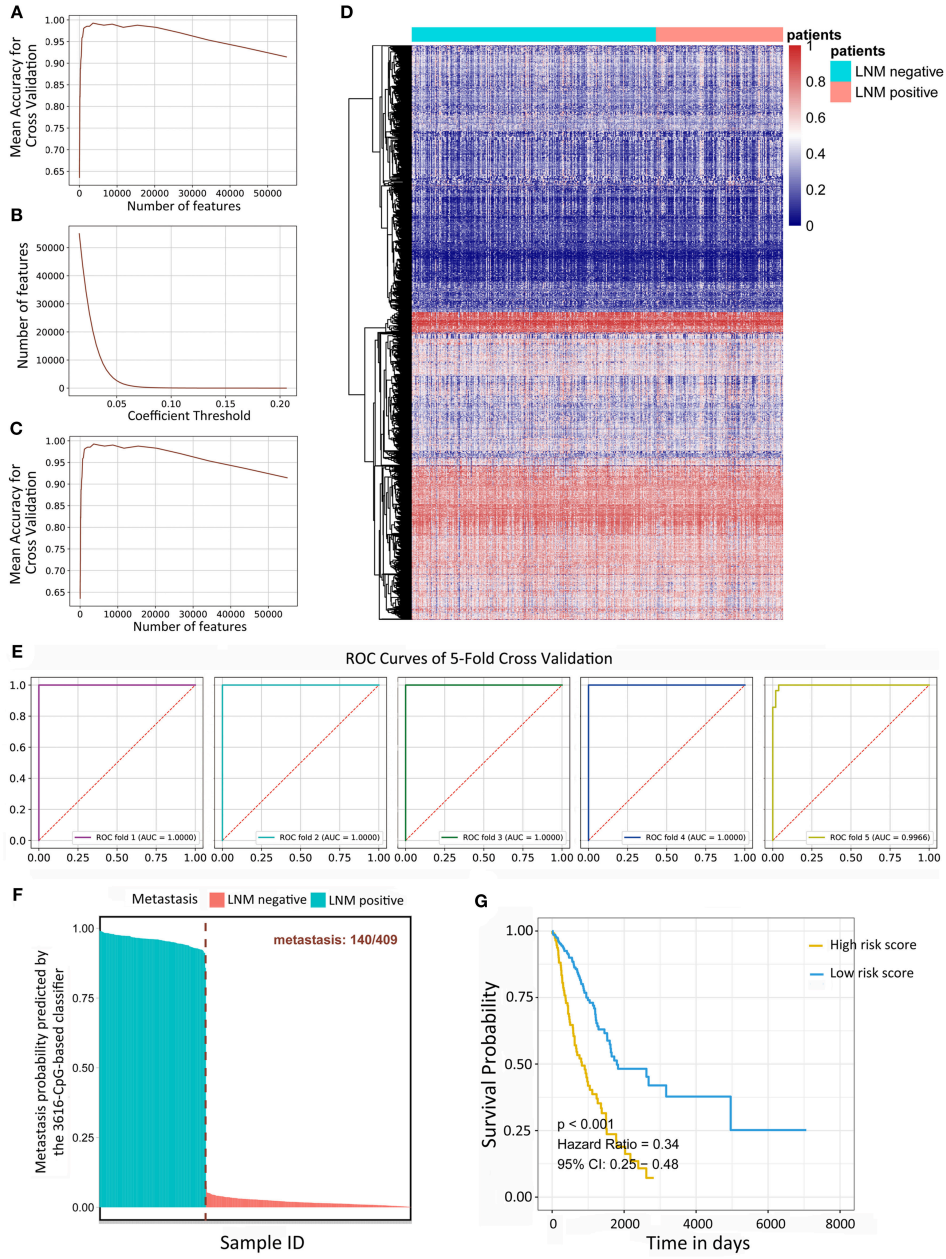
Linear logistic classifier using a 5-fold cross-validation for the metastatic analysis and the efficiency for the 3616-CpG-based classifier. **(A)** The mean accuracy trend for the number of features and mean accuracy for the cross-validation. **(B)** The methylation features with regard to the different values of coefficient thresholds. **(C)** Recursive feature elimination with a cross-validation test. **(D)** Unsupervised hierarchical clustering and heat map associated with the methylation profile (according to the color scale shown) to recognize metastasis in LUAD. **(E)** ROC curves for 5-fold cross-validation showing the high sensitivity and specificity of the classifier in predicting metastasis. **(F)** The metastatic probability of each sample calculated by coefficients of the methylation signature. **(G)** Kaplan-Meier survival analysis for LUAD patients, which are divided into low-risk and high-risk groups using a cutoff value of 0.5. LNM: lymph node metastasis.

Variance-based selection was applied to filter features (methylation CpG sites). Features with small variances tend to be less discriminative, so we filtered out features with a standard variance smaller than 0.01 and 135,094 methylation signatures were selected. Regularized logistic regression and cross-validation were then applied to weigh the importance of each feature. The 428 LUAD samples were randomly assigned to a test set or a validation set by the cross-validation method. In short, five rounds of cross-validation were performed using different partitions and the validation results were combined over five rounds to overcome overfitting. By varying the value of the coefficient threshold, we obtained a different number of features that could be kept. When we used those kept features to regress the linear Logistic model by 5-fold cross-validation, the mean accuracy trend was as follows (Figure 1A). The number of kept features with regard to the different values of coefficient thresholds was shown in Figure 1B. The best performance was achieved at the threshold value 0.05 with 6,198 features kept with a 5-fold cross-validation. Recursive feature elimination with the same cross-validation configuration was tested and the result indicated that the kept features were the optimum minimal set of all the features (Figure 1C). The value of kept methylation CpG sites was shown in Figure 1D. We assessed the accuracy of the 3616-CpG-based classifier for detecting metastasis with a ROC analysis (Figure 1E) with the same cross-validation configuration, and averaged the weights of selected features across different set as the final coefficients. Furthermore, the metastatic probability of each sample were calculated by the coefficients of kept methylation CpG sites (Figure 1F and Supplementary File 5). The AUC for the classifier achieved values close to 1in all of the 5-fold cross-validation, indicating the robust classifier effect. The tumor tissues in total dataset were divided into high metastatic risk score and low metastatic risk score groups, respectively, using 0.5 as the cutoff. The patients in the low metastatic risk score group have a longer OS than those in the high metastatic risk score group in the total datasets (*p* < 0.0001, Figure 1G) as well as in the separated 5-fold training and validation sets (*p* < 0.05, Supplementary Figure 1). We assessed the prognostic accuracy of the 3616-CpG-based classifier metastatic classifier with a time-dependent ROC analysis at varying follow-up times (500, 1,000, 1,500, 2,000, 2,500, 3,000 days) (Supplementary Figure 2). The accuracy was all around 66%, indicating that the 3616-CpG-based classifier for identifying metastasis could also work well for predicting the OS of LUAD patients.

### Identification of 14-CpG-Based Signature to Recognize Tumor and Normal Tissues

**Figure 2:**
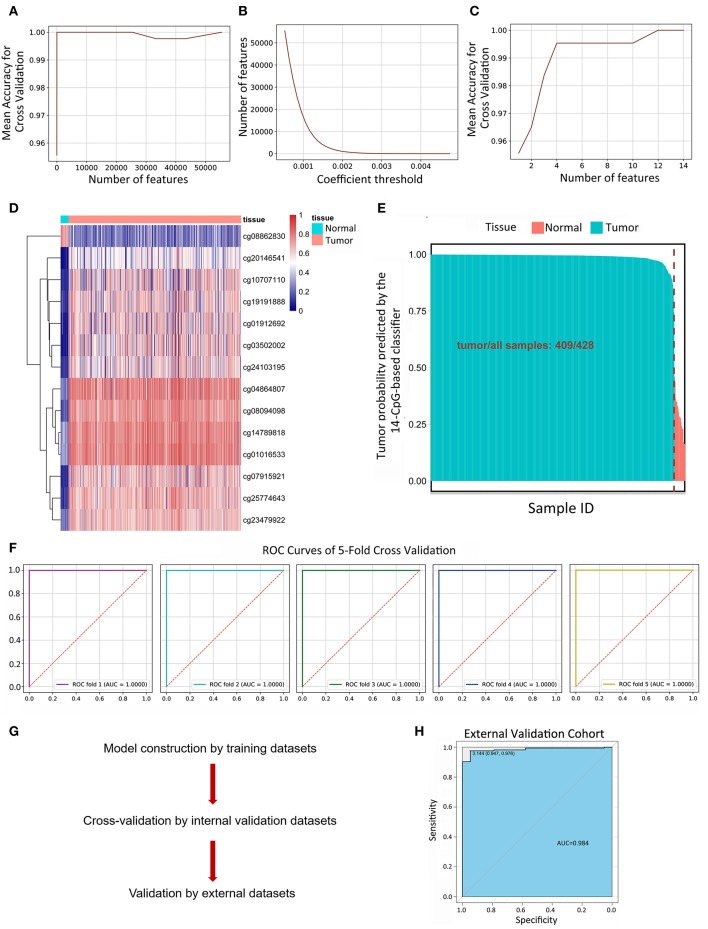
Linear logistic classifier using a 5-fold cross-validation to differentiate between LUAD tumor tissues and corresponding normal tissues, and the efficiency for the 14-CpG-based classifier. **(A)** The mean accuracy trend for the number of features and mean accuracy for the cross-validation. **(B)** The methylation features with regard to the different values of coefficient thresholds. **(C)** Recursive feature elimination with a cross-validation test. **(D)** Unsupervised hierarchical clustering and heat map associated with the methylation profile (according to the color scale shown) to differentiate between LUAD tumor tissues and corresponding normal tissues. **(E)** The probability of being tumor for each sample calculated by the coefficients of methylation signatures. **(F)** ROC curves showing the high sensitivity and specificity in differentiating between LUAD tumor tissues and normal tissues. **(G)** The workflow of model construction, internal validation and external validation. **(H)** ROC curve showing the high sensitivity and specificity in differentiating between LUAD tumor tissues and corresponding normal tissues on an external dataset.

134,015 features were kept by variance thresholding (0.01). Regularized Logistic regression and cross-validation were applied to weigh the importance of each feature as mentioned above. An accuracy of 100% can easily be achieved for the number of features range from 14 to 43,246 (Figure 2A). The number of kept features with regard to the different thresholding values was shown in Figure 2B. Recursive feature elimination with cross-validation was tested and the result indicated that an accuracy of 100% can be achieved when the kept feature numbers reached 14 (Figure 2C). 14 CpG sites were kept: cg25774643, cg03502002, cg14789818, cg23479922, cg04864807, cg07915921, cg20146541, cg08862830, cg01016533, cg19191888, cg08094098, cg01912692, cg10707110, cg24103195. The value of kept methylation CpG sites was shown in Figure 2D. We then calculated the probability of being tumor for each sample by the coefficients of kept methylation CpG sites (Supplementary File 6 and Figure 2E) in the same tradition as of the recognition of metastasis. The accuracy of the 14-CpG-based classifier was assessed by means of ROC analysis (Figure 2F). The results showed that the accuracy reached 100% in all 5-fold cross-validation, indicating the high sensitivity and specificity of the 14-CpG-based classifier in differentiating between LUAD tumor tissues and corresponding normal tissues. Furthermore, we applied the 14-CpG-based classifier on an external dataset to confirm the accuracy of the 14-CgG-based classifier (Figure 2G). The AUC value was 98.4% for differentiating the tumor and normal tissues (Figure 2H). The analysis before showed the regularized logistic model we applied worked well in different datasets.

### Identification of 16-CpG-Based Signature to Predict the OS of LUAD Patients

**Figure 3:**
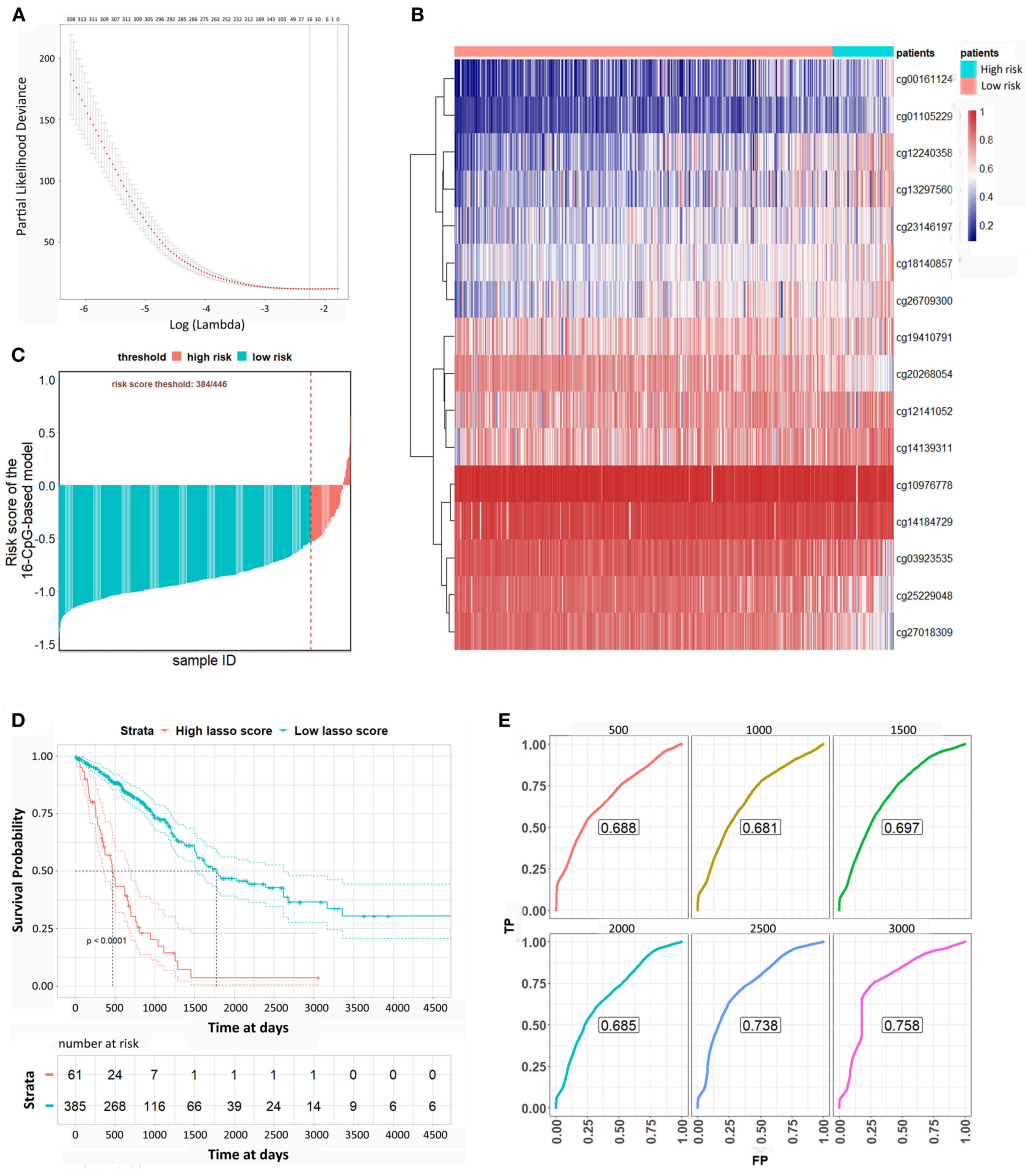
Lasso Cox analysis to predict the OS of LUAD patients. **(A)** The selection of λ value for Lasso Cox regression. **(B)** Unsupervised hierarchical clustering and heat map associated with the methylation profile (according to the color scale shown) to predict the OS of LUAD patients. **(C)** The risk scores of each sample calculated by the coefficients of methylation signatures from Lasso Cox analysis. **(D)** Kaplan-Meier curves of LUAD patients with a low or high risk of death, according to risk scores from the 16-CpG-based classifier. **(E)** Time-dependent ROC analysis at varying follow-up times (500, 1,000, 1,500, 2,000, 2,500, 3,000 days). We used AUC values at 500, 1,000, 1,500, 2,000, 2,500, 3000 days to assess the prognostic accuracy.

We used a LASSO Cox regression to build a prognostic model, which selected 16 methylation CpG sites from the CpG sites identified by the DNA methylation 450 k chip: cg00161124, cg01105229, cg03923535, cg10976778, cg12141052, cg12240358, cg13297560, cg14139311, cg14184729, cg18140857, cg19410791, cg20268054, cg23146197, cg25229048, cg26709300, cg27018309 (Figures 3A,B). The values of the 16 methylation CpG sites for each patient were shown in Figure 3B. A formula was derived to calculate the risk score for every patient based on their individual 16 methylation β values (Supplementary File 7). The risk scores of tumor samples were calculated by the coefficients of the kept methylation CpG sites (Figure 3C). The patients were divided into high risk score and low risk score groups, respectively, with a cutoff of −0·54. Kaplan-Meier survival analysis (Figure 3D) showed that the survival probability of patients in lower risk score was significantly better than in high risk score group (log-rank test, all *p* < 0.0001). We assessed the prediction accuracy of the 16-CpG-based model by means of time-dependent ROC analysis at varying follow-up times. The AUC values for 500, 1,000, 1,500, 2,000, 2,500, and 3,000 days were 0.688, 0.681, 0.697, 0.685, 0.738, and 0.758, respectively, which confirmed the effectiveness of the 16-CpG-based model to predict the OS of LUAD patients (Figure 3E).

**Figure 4:**
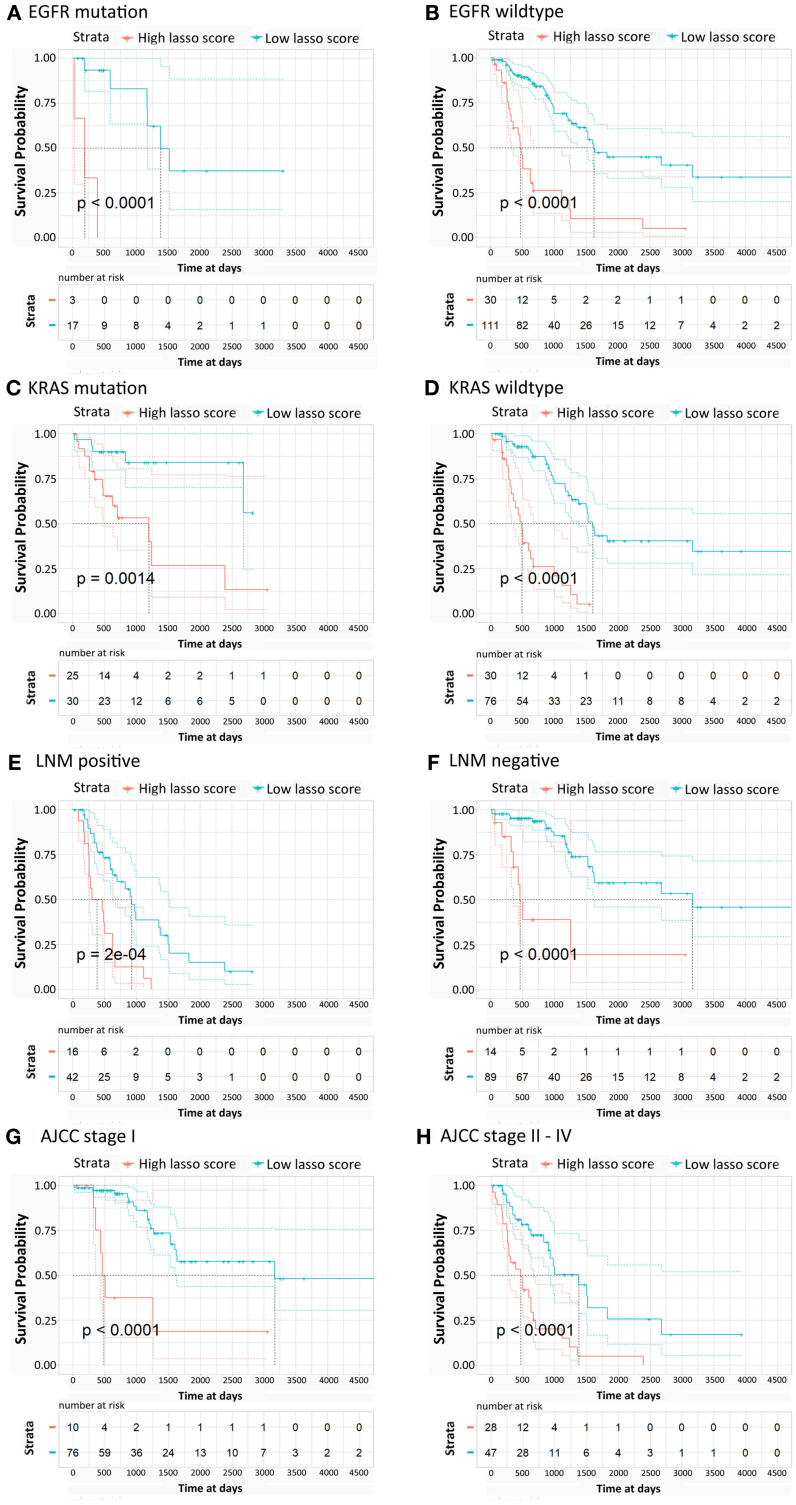
Kaplan-Meier survival analysis for LUAD patients according to the 16-CpG-based classifier. Patients were classified according to clinicopathological risk factors. **(A,B)** EGFR status; **(C,D)** KRAS status; **(E,F)** lymph node metastatic (LNM) status; **(G,H)** AJCC stage I and II-IV. The patients were divided into low-risk and high-risk groups. *P*-values were calculated using the log-rank test.

According to their clinicopathological conditions, like epidermal growth factor receptor (EGFR) mutation, K-ras or Ki-ras (KRAS) mutation, lymph node metastatic (LNM) condition, and AJCC stage, LUAD patients were divided up into several subgroups to validate the independent diagnostic value of the methylation signature. EGFR mutation showed a striking correlation with LUAD patient characteristics, which were correlated with the clinical treatment response and then affected the OS of LUAD patients. The Kaplan-Meier curves regarding EGFR mutation and wildtype groups were shown in Figures 4A,B. Patients with low risk scores generally had significantly better survival than those with high risk scores in both groups (*p* < 0.0001). Similarly, patients with low risk scores had a significantly longer OS than those with high risk scores in both KRAS mutation and wildtype subgroup and both LNM positive and negative groups (Figures 4C–F, *p* < 0.0001). For the patients in AJCC stage I and AJCC stage II-IV, the survival probability of patients with low risk scores was higher than those with low risk scores (Figures 4G,H). The stratification analysis above revealed that the 16-CpG-based model could effectively predict the OS of patients regardless of the patients' clinicopathological properties, and provide prognostic power to complement the clinical stage and SSIGN scores.

**Figure 5:**
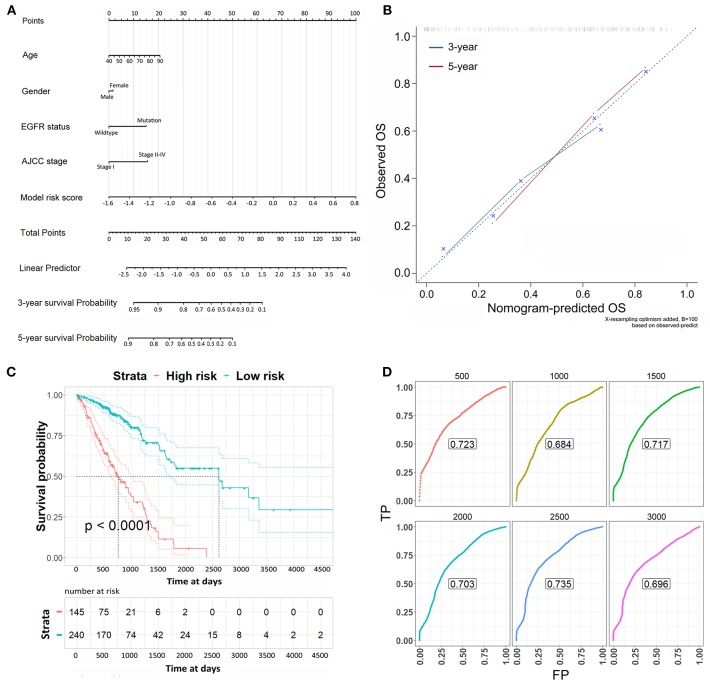
The nomogram to predict the risk of death in 3 and 5 years of LUAD patients. **(A)** The nomogram to predict the risk of death in 3 and 5 years of LUAD patients. **(B)** Plots depict the calibration of each model in terms of agreement between predicted and observed 3- and 5-year outcomes. The dashed line represents the ideal line of a perfect match between nomogram-predicted and observed OS. The blue and red line indicate the performance of the proposed nomogram for 3 and 5 years, respectively. **(C)** Kaplan-Meier survival analysis for the OS of LUAD patients according to the risk scores from the nomogram. **(D)** Time-dependent ROC curves from the nomogram for overall survival in 3 and 5 years.

Lastly, the risk scores were applied to the Cox regression model with the clinicopathological risk factors to perform multivariable survival analysis, thereby generating a nomogram to predict patients' survival probability for 3 and 5 years (Figure 5A). In the multivariable survival analysis, we included age, gender, EGFR status, AJCC stage, and risk scores from 16-CpG-based model. The nomogram was further verified with calibration plots (Figure 5B). The results showed that the nomogram fared well with the ideal mode for 3 and 5 years, indicating the nomogram worked well in predicting the OS of LUAD patients. According to the risk scores from the nomogram, patients were divided into high risk and low risk group. Kaplan-Meier survival analysis showed that the survival probability of patients with low risk score was significantly higher than those with high risk score (Figure 5C, log-rank test, *p* < 0.0001). The prognostic accuracy of the nomogram was further accessed by time-dependent ROC curves (Figure 5D). The results showed that the AUC values were all around 0.7 at varying follow-up times (500, 1,000, 1,500, 2,000, 2,500, 3,000 days), indicating the high effectiveness of the nomogram in predicting the prognostic OS of LUAD patients.

## Discussion

The present study demonstrates the potential of using DNA methylation signatures to identify the lymph node metastasis of primary LUAD tissues, to differentiate between the LUAD tumor and normal tissues, and to predict the OS of LUAD patients. Invasive biopsy is the gold standard for the validation of tumor tissues and identification of histological subtypes. However, the collection of bulk tissue samples for immunohistochemical (IHC) staining may cause secondary damage to patients. An inadequate tissue yield or quality also creates barriers for the histological diagnosis. Besides, it may be difficult to identify lymph node metastasis during operation. Nowadays, molecular characterization methods provide new insights in pathological diagnosis (Tsou et al., 2007; Selamat et al., 2012; Zhang et al., 2013; Ogino et al., 2016). Since the global change of DNA methylation takes place at the beginning of carcinogenesis, DNA methylation has been considered a promising biomarker for the early detection and diagnosis of cancers (Franco et al., 2008; Hatano et al., 2015; Wu and Ni, 2015), which can complement the pathological IHC staining. Moreover, DNA methylation analysis does not require bulk tissue samples. Small amounts of tissue are enough for DNA extraction and methylation-chip or methylation-seq analysis, which will reduce the patients suffering. Hundreds of thousands of the DNA methylation CpG sites can be identified through genome-wide DNA methylation detection by DNA methylation chips or methylation-seq. Discovering a potential panel of DNA methylation-based biomarkers from the large DNA methylation files can be beneficial for the early diagnosis of cancer initiation and metastasis. Several research studies have shown the potential of utilizing DNA methylation profiles to help the diagnosis of different cancers (Diaz-Lagares et al., 2016; Zhang et al., 2017; Sandanger et al., 2018). One study applied an unsupervised clustering method on DNA methylation profiles to find potential subtypes of childhood B-cell acute lymphoblastic leukemia. The patients were allocated into two subgroups by the unsupervised hierarchical clustering of DNA methylation profiles, which showed a significant association between DNA methylation and disease-free survival (Sandoval et al., 2013a). Another study also utilized a similar strategy to find the association between DNA methylation signatures and the recurrence-free survival in non-small-cell lung cancer samples (Sandoval et al., 2013b). In our study, we applied supervised learning strategy (regularized logistic regression) to find the prognostic CpG cites in LUAD primary tissues. The RFE helped to eliminate the unnecessary features in regression, which constrained the numbers of key CpG sites for prognosis. Besides, LASSO Cox regression was useful to reduce the feature numbers in the COX survival analysis. One study built a prognostic signature by LASSO Cox regression to predict the progression-free survival of LUAD patients and demonstrated the potential biological significance of DNA methylation in the etiology of LUAD (Bjaanæs et al., 2016). Another study built a mortality risk score by LASSO Cox regression (Zhang et al., 2017). The signature based on ten selected CpG sites exhibited strong association with all-cause mortality. Moreover, one recent study used blood-derived DNA methylation and gene expression profiles to identify CpG lung cancer markers prior to diagnosis. They emphasized the difference of prognostic CpG sites in smoking and non-smoking lung cancer patients (Sandanger et al., 2018). In this study, based on the methylation profiles of LUAD patients, we performed regularized logistic regression and LASSO Cox regression to identify the lymph node metastasis, to differentiate between tumor and normal tissues and to predict the OS of LUAD patients. From the primary LUAD tumor tissues, 3616 methylation CpG sites were kept to build a classifier to identify LUAD lymph node metastasis. ROC curves showed the high sensitivity and specificity of the 3616-CpG-based classifier in identifying lymph node metastasis from CpG sites of primary tumor tissues. All the samples came from the primary tumor tissues, which means that the metastatic behavior can be identified even without extracting tissues from lymph nodes. Therefore, it would work as a biomarker to predict the diagnosis of lymph node metastasis. Since the metastatic behavior of LUAD affects the OS of LUAD patients dramatically, we applied the metastatic classifier to check whether the model can be used to predict the OS of LUAD patients. The time-dependent ROC curves showed the effectiveness of the metastatic classifier in predicting the OS of LUAD patients at varying follow-up times. As expected, the patients in the high metastatic risk score group have a significantly worse OS than those in the low metastatic risk score group.

Tumor tissues are heterogeneous tissues that include cancer cells (epithelial cells), cancer stem cells, vascular epithelial cells and so on (Reya et al., 2001; Marusyk et al., 2012). More than 70% of the tumor tissues are cancer cells. The heterogeneity of tumor tissues may influence the accuracy of the diagnosis. We compared the heterogeneous tumor tissues with the normal tissues, which also include the vascular epithelial cells and other cell types. Considering the heterogeneous tumor tissues and heterogeneous normal tissues as a whole for each, we tried to eliminate the influence brought about by heterogeneity (Li et al., 2014). In this study, to differentiate between tumor and normal tissues, we concluded that 14 CpG methylation sites were enough for the diagnosis. To check the overfitting potential, we applied 5-fold cross-validation. The efficiency of the model above was tested by a ROC curves in five different training and validation datasets, which showed the high efficiency and specificity of the 14-CpG-based classifier in differentiating between LUAD tumor tissues and the normal tissues. Furthermore, we also validated our regression model on the external dataset from one study (Bjaanæs et al., 2016). Results showed an AUC value of 98.4% to differentiate the tumor and normal tissues by the ROC analysis. The external dataset further confirmed the accuracy of the regularized logistic model which we applied to build the both classifiers above. From the two classifiers, we obtained an overlap cluster of CpG sites: cg03502002 and cg07915921. The information for cg07915921 is not clear. cg03502002 is on the CpG island of the promotor region of the GALR1 gene. The methylation status of the GALR1 promoter and the level of GALR1 gene expression have been correlated in a large number of head and neck squamous tumor specimens (Misawa et al., 2008). Ectopic expression of GALR1 suppresses tumor cell proliferation through Erk1/2-mediated regulation of cyclin-dependent kinase inhibitors and cyclin D1 (Kanazawa et al., 2009). One study revealed that hypermethylated GALR1 plays important roles in smoking-associated LUAD (Tan et al., 2013).

We also built a model to predict the OS of LUAD patients by means of methylation CpG sites. The LASSO Cox regression model generated risk score for each patient. When we assessed the survival status and distribution of risk scores, patients with low risk scores generally had a better OS than those with high risk scores. The model will help guide individualized follow-up schedules for LUAD patients. The high-risk patients have poor OS prediction. This could be the basis of a future clinical trial. The LASSO Cox regression results were further confirmed by the time-dependent ROC analysis. When we compared the time-dependent ROC from the OS-prediction model and metastasis-prediction classifier, the OS-prediction model turned out to be more precise in the long-term survival prediction while the metastasis-prediction classifier worked better in the short-term survival prediction. One explanation could be that when the LUAD patients were accompanied by lymph node metastasis, the tumor progressed and the patients had a poorer prognosis. The OS expectation of patients with lymph node metastasis was shorter than those without lymph node metastasis. Hence, the metastasis-prediction classifier would work better for the short-term prediction.

To further utilize the risk scores from the Cox regression model, we classified patients into several subgroups according to the clinicopathological risk factors (EGFR mutation, KRAS mutation, LNM status and AJCC stages). The 16-CpG-based classifier still showed clinical and statistical significance regardless of the clinicopathological status of LUAD patients.

The independent prognostic values of the 16-CpG-based model were validated by multivariable survival analysis, which integrated other clinicopathological risk factors for the OS of LUAD patients. The Cox regression risk scores were applied together with age, gender, EGFR status, AJCC stages as indicators to generate a nomogram to predict the 3- and 5-year survival probability. We verified the performance of the nomogram by calibration plots. The predicted OS of LUAD patients by the nomogram was highly consistent with the observed 3- and 5-year OS of LUAD patients. Log-rank test and time-dependent ROC curves at vary follow-up times further confirmed the nomogram. Thus, the nomogram could provide an accurate and simple prognostic prediction for LUAD patients.

In previous studies, mRNA expression files (Beer et al., 2002), the mutation of key genes (Takano et al., 2008; Kosaka et al., 2009), long no-coding RNA expressions (Kosaka et al., 2009; Huarte, 2015; Zhou et al., 2016), and histone modifications (Seligson et al., 2009; Zhou et al., 2016) showed the prognostic potential for different types of cancer. Here, we emphasized that the methylation patterns could also be a meaningful tool for the prognosis of LUAD patients. Some studies have identified that multiple CpG sites are differentially methylated in lung cancer compared to normal tissues (Genome Atlas Research Network., 2014; Poirier et al., 2015; Hao et al., 2017). The key for methylation pattern-based early diagnosis is the identification of crucial CpG sites in LUAD. The use of supervised machine learning methods allowed us to integrate all methylation CpG sites identified by the methylation chip into one model, which improved the prognostic accuracy over that of a single CpG site alone. Our findings show that three DNA CpG signature-based models can effectively identify lymph node metastasis by the CpG sites from primary tumor tissues, differentiate between tumor and normal tissues, and predict the OS of LUAD patients. The tissues would be collected by preoperative biopsy or at surgery. The classifiers for identifying lymph node metastasis and differentiation between tumor and normal tissues would help the preoperative diagnosis. The Lasso Cox model would be helpful for adjuvant treatment and prognostic planning. Therefore, the 3 methylation signatures could be of great value in assessing the status, predicting prognosis and achieving individualized treatments of LUAD patients.

The limitations of our study should be mentioned. The methylation 450 k chip did not identify as many CpG sites as the methylation 850 k chip or methylation sequencing. The methylation CpG site candidates identified here did not represent the complete CpG sites in the genome of LUAD patients.

In conclusion, we built three DNA CpG signature-based models to identify LUAD lymph node metastasis by the CpG sites from primary tumor tissues, differentiate between tumor tissue and normal tissue, and predict the OS of LUAD patients, which highlight the relationship between clinical results (metastasis, survival) and methylation biomarkers in LUAD patients. The nomogram comprising LASSO Cox risk scores and clinicopathological factors may help predict the OS of LUAD patients and help individualized treatment of LUAD patients.

## Author Contributions

XB and RS conceived and designed the experiments. XB and HD wrote the code (The Python code is available due to request). XB, YW, and SX wrote the paper. RS and KZ reviewed the manuscript. All authors read and approved the final manuscript.

### Conflict of Interest Statement

The authors declare that the research was conducted in the absence of any commercial or financial relationships that could be construed as a potential conflict of interest.

## Supplementary Material

Supplementary Figure 1 Kaplan-Meier survival according to risk scores from the 3616-CpG-based classifier in the training, validation sets for 5-fold cross-validation. **(A, C, E, G, I)** The training sets 1-5. **(B, D, F, H, J)** The validation sets 1-5.

Supplementary Figure 2 Time-dependent ROC analysis at varying follow-up times (500, 1,000, 1,500, 2,000, 2,500, 3,000 days) according to risk scores from the 3616-CpG-based classifier.
