# Supplementary figures and images for: Prognostic and Predictive Value of Three DNA Methylation Signatures in Lung Adenocarcinoma
Source: Front Genet. 2019 Apr 24;10:349. doi: 10.3389/fgene.2019.00349 (PMC6492637; doi:10.3389/fgene.2019.00349)

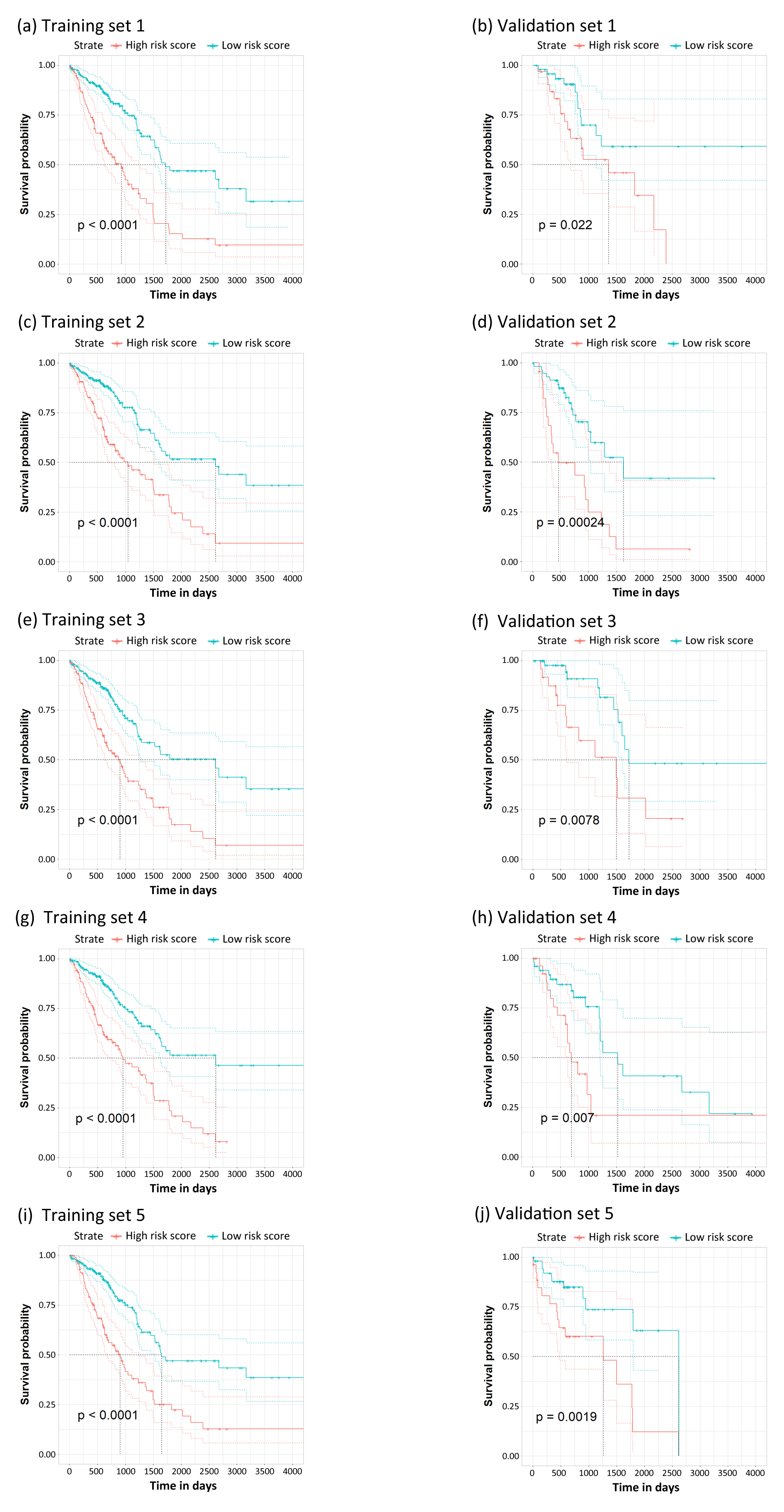

Supplement: Supplementary Figure 1 — Kaplan-Meier survival according to risk scores from the 3616-CpG-based classifier in the training, validation sets for 5-fold cross-validation. (A, C, E, G, I) The training sets 1-5. (B, D, F, H, J) The validation sets 1-5. [file Image_1.TIF]

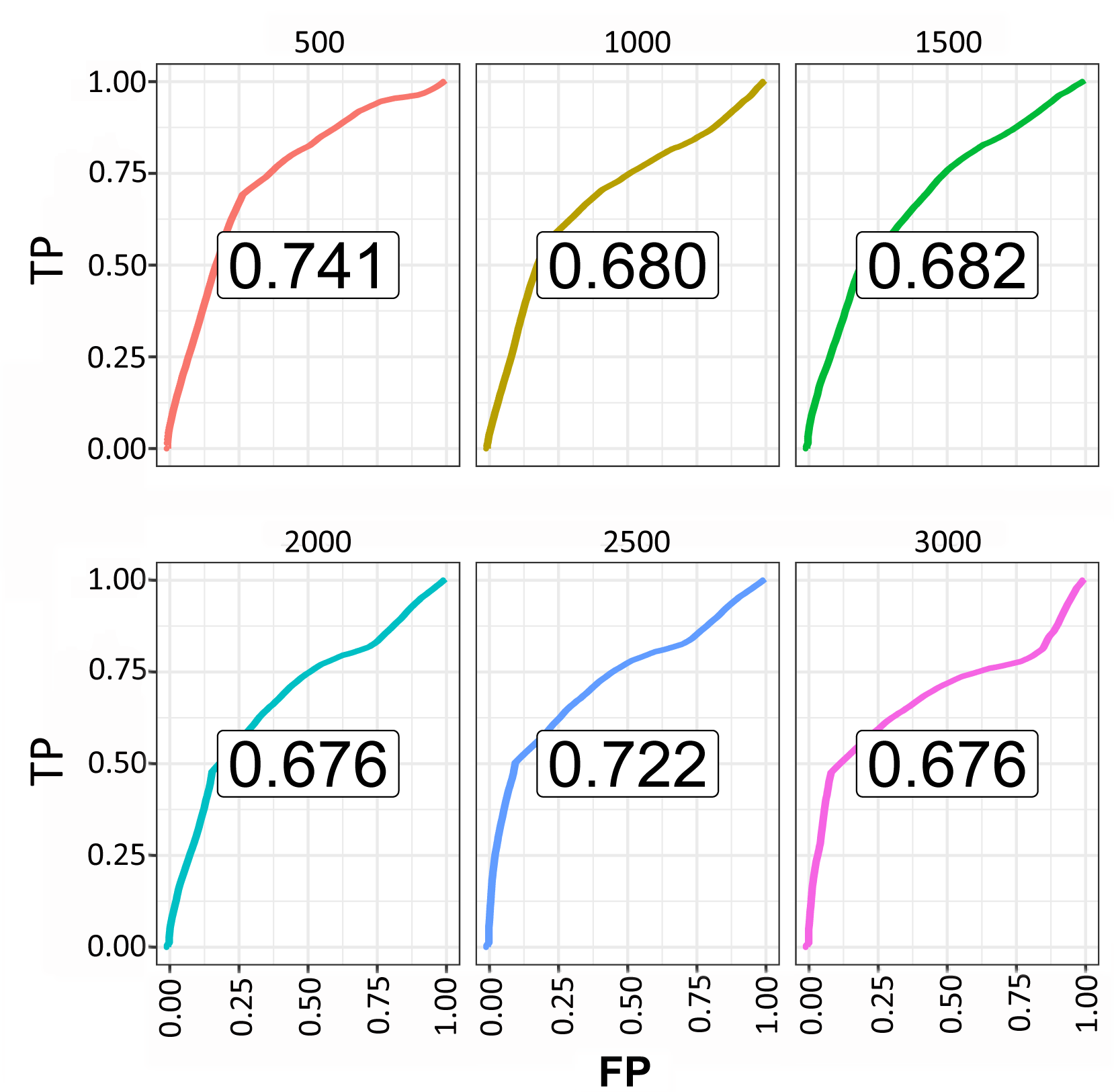

Supplement: Supplementary Figure 2 — Time-dependent ROC analysis at varying follow-up times (500, 1,000, 1,500, 2,000, 2,500, 3,000 days) according to risk scores from the 3616-CpG-based classifier. [file Image_2.TIF]
